# Effect of Nigella Sativa Oil on Oxidative Stress, Inflammatory, and Glycemic Control Indices in Diabetic Hemodialysis Patients: A Randomized Double-Blind, Controlled Trial

**DOI:** 10.1155/2022/2753294

**Published:** 2022-04-15

**Authors:** Alireza Rahmani, Bahram Niknafs, Mohsen Naseri, Maryam Nouri, Ali Tarighat-Esfanjani

**Affiliations:** ^1^Student Research Committee, Student Research Center, Tabriz University of Medical Sciences, Tabriz, IR, Iran; ^2^Department of Internal Medicine, School of Medicine, Imam Reza Medical Research and Training Hospital, Tabriz University of Medical Sciences, Tabriz, IR, Iran; ^3^Traditional Medicine Clinical Trial Research Center, Shahed University, Tehran, Iran; ^4^Department of Nutrition Sciences, Varastegan Institute for Medical Sciences, Mashhad, IR, Iran; ^5^Nutrition Research Center, Clinical Nutrition Department, Faculty of Nutrition and Food Sciences, Tabriz University of Medical Sciences, Tabriz, Iran

## Abstract

**Background and Aims:**

Diabetes is a leading cause of renal failure. High levels of oxidative stress and inflammation in patients with renal diabetes lead to various disorders and mortality. This study was performed to determine the effect of Nigella sativa (NS) supplementation on superoxide dismutase (SOD), malondialdehyde (MDA), total antioxidant capacity (TAC), high-sensitivity C-reactive protein (hs-CRP), glycosylated hemoglobin (HbA1c), fasting blood sugar (FBS), and insulin (INS) in patients with diabetes mellitus undergoing hemodialysis (HD).

**Methods:**

In this randomized, double-blind, placebo-controlled clinical trial, a total of 46 diabetic HD patients were randomly divided into NS (*n* = 23) and placebo (*n* = 23) groups. NS group received 2 g/day of NS oil, and the placebo group received paraffin oil for 12 weeks. Serum levels of SOD, MDA, TAC, hs-CRP, HbA1C, FBS, and INS were measured before and after the study.

**Results:**

Compared to baseline values, SOD, TAC, and INS levels increased, whereas MDA, hs-CRP, HbA1c, and FBS significantly decreased. After adjusting for covariates using the ANCOVA test, changes in the concentrations of SOD (*p* = .040), MDA (*p* = .025), TAC (*p*=<.001), hs-CRP (*p* = .017), HbA1c (*p* = .014), and FBS (*p* = .027) were statistically significant compared to the placebo group. Intergroup changes in INS were not significant. Additionally, there were no notable side effects during the research.

**Conclusions:**

This study found that NS supplementation significantly enhanced the levels of SOD, MDA, TAC, hs-CRP, HbA1c, and FBS in diabetic HD patients.

## 1. Introduction

Diabetes mellitus (DM) is a leading cause of renal failure, resulting in end-stage renal disease (ESRD). Dialysis has been the primary treatment method for ESRD patients for nearly 60 years [1]. Half of the dialysis patients are diagnosed with diabetes [2]. The prevalence of diabetes and renal failure is increasing, and currently, about 2 million patients annually undergo dialysis [3]. Although dialysis improves the quality of life, ESRD patients are susceptible to many complications and disabilities. The prevalence of myocardial infarction, stroke, infections, depression, and the mortality rate in this group is higher than the general average [4], which is attributed to high oxidative stress (OS) and inflammation existing in these people [5–7].

The OS is defined as the overproduction of reactive oxygen species (ROS) and has overwhelming effects on the antioxidant defense system. It has been considered a significant hallmark for the pathogenesis and development of type 2 DM (T2DM) [8]. Various mechanisms are involved in generating ROS. A high-glucose environment in DM triggers protein glycation and oxidation. The glycated proteins are further modified and oxidized to release free radical products, the advanced glycation end products (AGEs).

Moreover, lipid peroxidation has been suggested as a significant causative factor for the development of OS [9]. HD might exacerbate the disease by increasing OS production. Some reasons for this claim include comorbidities that usually accompany HD, impaired antioxidant defense mechanisms, dietary limitation, and blood exposure to dialyzer membranes [5].

Another major problem of these patients is their inflammatory status. Several studies have indicated an inseparable link between OS and general inflammation [10]. There was a positive relationship between elevated serum CRP levels and lipid peroxidation in an HD patient group. Oxidative metabolites, such as hydrogen peroxide, can activate the nuclear factor kappa light chain enhancer of the activated B-cell (NF-kB) pathway, promoting the synthesis of proinflammatory cytokines and amplifying the inflammatory cascade [6,11]. Therefore, oxidant molecules contribute to renal damage by stimulating renal ischemia, glomerular injury, apoptosis, and, finally, stimulating a severe inflammatory process [12].

As a result, during the last two decades, OS and consequent inflammation have become the center of attention as a novel, nontraditional risk factor for atherosclerosis, DM, and CKD progression [5,6]. Administration of antioxidants such as vitamin *E* and vitamin C in HD patients is example of this claim in previous studies [6,13]. Although the administration of antioxidants appears to play a beneficial role in OS development in the maintenance of HD patients, more documentation and clinical practice are needed [12].

Herbal medicines to cure ailments have received much attention because of their relatively low cost, limited side effects, and significant efficacy. Nigella sativa (NS), commonly known as black seed, is a member of the Ranunculaceae family of plants. It has been used in traditional medicine to treat various ailments, including rheumatoid arthritis, DM, gastrointestinal disease, hypertension, dyslipidemia, and immune deficiency diseases [14–18]. The main active compounds of NS include thymoquinone (TQ), nigellicine, nigellidine, thymohydroquinone, carvacrol, tocopherol (*α*, *β*, and *γ*), retinol, *ß*-carotene, *a*-hederin, phytosterols, and thymol [19,20]. Thymoquinone is a well-studied NS compound. Previous research studies have linked this ingredient to various medicinal benefits (antioxidant, anti-inflammatory, antihyperglycemic, anticancer, and antihistaminic) [21].

Considering the positive effects of NS supplementation as an antioxidant and anti-inflammatory agent in many diseases, and the high levels of OS and inflammation in DM patients treated by HD, and because no clinical trials have determined this issue, this study aimed to investigate the efficacy of NS in the status of OS and inflammation in these patients.

## 2. Materials and Methods

### 2.1. Study Design and Participants

This study is a randomized, double-blinded placebo-controlled, three-month, parallel-group clinical trial. It was conducted at Imam Reza Hospital, Tabriz University of Medical Sciences, Tabriz, Iran. Diabetic HD patients were recruited as study participants. They were interviewed, and a questionnaire concerning demographics, medication use, and smoking status was completed. The inclusion criteria were as follows: age from 20 to 60 years old, body mass index (BMI) from 18.5 to 30 kg/m^2^, three HD sessions per week, six months on HD, and willingness to participate in the study. Exclusion criteria were pregnancy or lactation, cigarette smoking, substance abuse, cancer, hepatic, and thyroid disorders; taking nonsteroidal anti-inflammatory (NSAID) or cytokine inhibitor drugs; antioxidant supplements two months before or during the study; and regular use of NS oil. Those who had less than 90% adherence or changed their normal medicines and diet during the intervention were eliminated.

### 2.2. Intervention

For 12 weeks, participants in the intervention group were given two g/d of NS oil soft gel capsules (one capsule, twice daily). In contrast, those in the placebo group were given the same amount of paraffin oil. Barij Essence Pharmaceutical Company manufactured the supplements using the cold press technique (Kashan, Iran). Both NS oil and paraffin oil capsules were packaged in dark containers with similar colors, smells, and appearances. Each container, including 30 capsules, was given to patients once every two weeks, and they were asked to take it after dialysis and at intervals from meals. The dose was determined based on Kaatabi H [22], which is among the most secure and effective NS oil supplementation in diabetic patients.

### 2.3. Follow-Up

All patients were requested not to change their physical activity and diet during the study. Participants were visited in the HD center every two weeks and monitored for any probable adverse events. To ensure compliance with the intervention protocol, they were asked to return the previous empty bottle when a new one was delivered. The researcher's phone number was provided to the patients for better follow-up.

### 2.4. Primary and Secondary Outcomes

The primary outcomes of this study were SOD, MDA, TAC, hs-CRP, HbA1c, fasting blood sugar (FBS), and insulin (INS). Secondary outcome measures were macronutrient and energy intake stats.

### 2.5. Biochemical Assessment

Following 10–12 hr of fasting, 10 ml of venous blood was collected from the participants at the beginning and end of the study. The serum samples were separated from whole blood by centrifugation at 3,000 rpm for 7 minutes (Orum Tadjhiz Centrifuge, Iran) at room temperature. Serums were stored at -70 °C until assay time.

Hs-CRP, SOD, MDA, TAC, INS, and HbA1c concentrations in patient's serum samples were measured using relevant commercially available diagnostic kits (Navandsalamat Co., Iran) according to the company package insert instruction. Patients were asked not to smoke or engage in physical activity for 30 minutes before blood sampling.

### 2.6. Anthropometric Indices

A stadiometer with 0.1 cm accuracy measured the participants' height standing near the wall. Weight was measured using a weighing scale (Seca, Hamburg, Germany) with minimal clothing and without shoes to the nearest 0.1 kg.

### 2.7. Sample Size Calculation

The determination of the sample size for this study was based on the primary data on creatinine outcomes obtained from the previous study (Z. Ansari, SJKDT, 2017 study) [23]. Assuming the power of 90% and the confidence interval of 95% and using the usual formula for clinical trials (((*σ*_1_^2^+*σ*_2_^2^)(*Z*_*α*/2_+*Z*_*β*_)^2^/(*μ*_1_ − *μ*_2_)^2^), the sample size was estimated at 21. Eventually, to cover 10% dropout, 23 patients were recruited in each group.

### 2.8. Randomization and Blinding

Patients were divided into two groups, including NS or placebo groups using random allocation software and in block sizes of four by a statistics professional. To control confounders, the individuals were properly matched based on HD frequency (2 or 3 times per week) and blood sugar levels (FBS< 120 mg/dL, FBS = 120–200 mg/dL, and FBS> 200 mg/dL). To keep participants and investigators blind until the end of the trial, a pharmacist initially classified NS and placebo bottles as A and B. There was no difference in appearance, color, or smell between the NS and the placebo capsules. The study's blinding code was not revealed until the end.

### 2.9. Statistical Analysis

The IBM SPSS software version 23 (IBM SPSS Statistics, Armonk, USA) was used to analyze the data. The Shapiro-Wilk test was used to assess the normality of data. Mean ± standard deviation (SD) and mean difference (95% CI) were reported to describe customarily distributed data. Median (IQB) and median differences were reported for non-normal variables. For normal and abnormal distribution data, independent samples *t*-test or Mann-Whitney *U* tests were, respectively, used to analyze differences between groups at baseline. As appropriate, the intragroup differences for normal and abnormal distribution values were analyzed using paired samples *t*-tests and Wilcoxon signed-rank tests. The analysis of covariance (ANCOVA) test was performed to exclude the influence of confounding variables (i.e., baseline values, energy intake, and BMI) on the results. The aim was achieved by employing two distinct models, one of which contained baseline data (model 1), and the other included baseline values, BMI, and calorie intake (model 2). A significance level of 0.05 was used for all analyses, and the final analysis did not include missing data.

### 2.10. Ethical Consideration

Volunteers filled out an informed consent form after the study protocol was explained to them. Under the Declaration of Helsinki, the ethics committee of Tabriz University of Medical Sciences (TBZMED), Tabriz, Iran, provided ethics approval (approval ID : IR.TBZMED.REC.1399.109). Also, the trial was registered on the Iranian Registry of Clinical Trials (registration number: IRCT20200411047027N1).

## 3. Results

**Figure 1:**
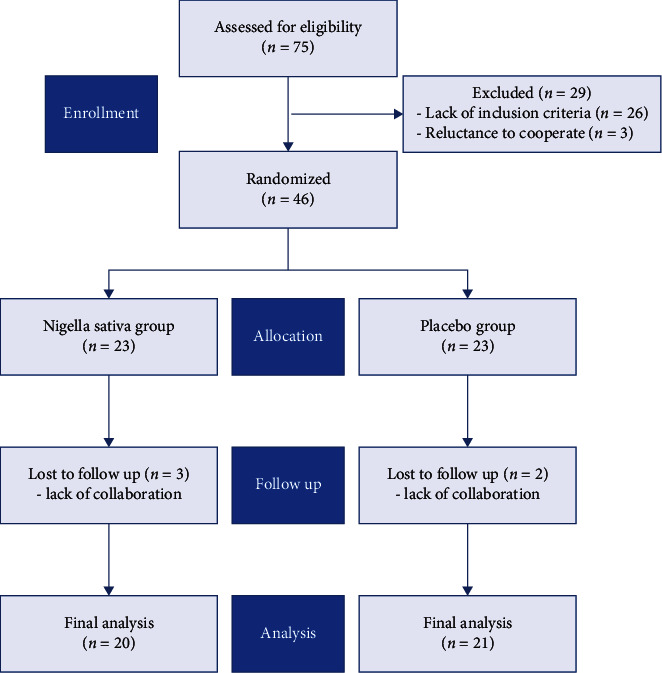
Flowchart of recruitment and randomization process.

Figure 1 shows the recruiting and randomization processes.

Table 1 displays the demographic information of both groups. There was no significant difference between the two groups at the start of the trial.

**Figure 2:**
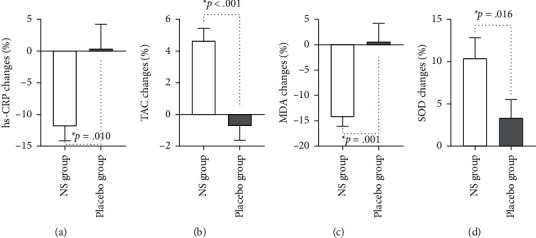
The effect of Nigella sativa on SOD, MDA, TAC, and hs-CRP in two study groups: (a) percentage of hs-CRP changes; (b) percentage of TAC changes; (c) percentage of MDA changes; and (d) percentage of SOD changes,  ^*∗*^ ANCOVA test (adjusted for BMI and energy intake). All the values are mean (SEM). SOD = superoxide dismutase; MDA = malondialdehyde; TAC = total antioxidant capacity; hs-CRP = high-sensitivity C-reactive protein; ANCOVA = analysis of covariance.

Table 2 shows the biochemical changes in the study. At the beginning of this study, there was no statistically significant difference between the two groups for any of the variables, except for MDA. At the end of the trial, the intergroup analysis of the intervention group revealed a substantial decrease in MDA, hs-CRP, and HbA1c and a significant rise in SOD, TAC, and INS. After adjusting for baseline values, energy intake, and BMI, an intragroup analysis indicated statistically significant changes in MDA, hs-CRP, SOD, TAC, HbA1c, and FBS but not in INS levels between groups. The percentage of SOD, MDA, TAC, and hs-CRP changes in the two groups significantly differed (Figure 2). During the research, there were no significant adverse effects.

## 4. Discussion

As far as we know, this is the first study to examine the effects of NS supplementation on patients with HD diabetes. According to the results of this clinical trial, NS oil caused significant changes in MDA, SOD, TAC, hs-CRP, FBS, and HbA1c indices compared to the placebo group. But between-group changes in INS were not significant.

Previous studies have reported the positive effects of NS supplementation on antioxidant and anti-inflammatory indices. Many animal studies have investigated this issue, but human studies are limited. In 2015, Huda Kaatabi et al. prescribed 2 g/d of NS powder to diabetic patients within a 48-week randomized clinical trial (RCT). Their analysis showed a significant decrease in thiobarbituric acid-reactive substances and increased SOD and TAC. These changes were also significant compared to the placebo group [22]. In this regard, Saeid Hadi et al. administered 1 g/d of Nigella oil for 8 weeks. Finally, MDA substantially decreased in the intervention group. However, SOD changes were not significant. No change in SOD can be attributed to the low dose and the short duration of the study [24]. Some human studies have also examined the anti-inflammatory effect of black seed.

In 2016, Mahdavi et al. prescribed 3 g/d of black seed oil along with a low-calorie diet to obese women. After 8 weeks, TNF-*α* and hs-CRP levels significantly decreased compared to the placebo group [14]. In another study by Kheirouri et al., daily consumption of 1 g of black seed oil by rheumatoid arthritis women showed a significant reduction in the hs-CRP level of the intervention group compared to the placebo group [25]. In 2019, Darand et al. administered black seed powder to nonalcoholic fatty liver disease patients for 12 weeks (2 g/d). Finally, intragroup analyzes reported significant changes for hs-CRP and TNF-*α*. However, intergroup changes in hs-CRP were not significant [26]. According to the totality of mentioned studies, it can be said that our results were in line with the previous trials. In addition, subgroup analyses have stated that NS oil has shown more anti-inflammatory effects than its powder.

Several mechanisms have been proposed concerning the anti-inflammatory effect of nigella sedan. The arachidonic acid (AA) metabolism pathway is one of the main pathways to produce inflammatory mediators in the body. Bioactive compounds in NS prevent the synthesis of inflammatory eicosanoids by inhibiting critical enzymes of this pathway. Phospholipase A2 (PLA2) is the first enzyme in this pathway to produce AA from membrane phospholipids. Saadat et al. showed that the carvacrol extracted from black seed with inhibition of PLA2 could show anti-inflammatory effects [27]. In addition, NS reduces 5HPETE by inhibiting the 5-lipoxygenase (the second key enzyme), thus reducing the synthesis of 2-series leukotrienes, which play an essential role in the body's inflammatory processes [28]. COX2 is the third key enzyme associated with the AA pathway inhibited by thymoquinone, which plays a crucial role in the inflammation process by synthesizing PGE2 and TXA2 [29]. Several mechanisms have been mentioned regarding the antioxidant effect of NS. The NADPH oxidase enzyme complex (NOX) is the primary source of ROS production in endothelial cells. TNF-*α* also increases ROS production by increasing P35, NOX1, and NOX2 [30, 31]. Previous studies have shown the inhibitory effect of thymoquinone on TNF-*α* and NOX [32, 33]. Moreover, superoxide anion (O_2_^−^) and hydrogen peroxide (H_2_O_2_) are the main compounds that can cause ROS to increase. SOD, catalase, glutathione peroxidase, and myeloperoxidase play a key role in combating ROS by converting hydrogen peroxide to H20. NS exerts its antioxidant effect by increasing SOD, GPx, and CAT, and decreasing MPO [32, 34–37]. Moreover, other mechanisms such as upregulation of anti-inflammatory hormone (like adiponectin) [38], reduction of IL-1*β* and IL-6, and increase in IL-10 are effective in creating anti-inflammatory and antioxidant effects of NS [39, 40].

Many studies have noted the antidiabetic effects of NS. In a placebo RCT study, Hosseini et al. administered 5 cc/d of NS oil daily to diabetic patients for 3 months and observed a significant reduction in FBS, postprandial blood glucose, and HbA1c [41]. In a similar study, Heshmati prescribed 3 g/d of NS oil in diabetic patients and reported a substantial reduction in INS resistance, FBS, and HbA1c after 3 months. However, the changes between groups of INS resistance were not significant after adjusting for confounder factors [42]. Kaatabi et al. administered 2 g/d of NS powder to diabetic patients for 48 weeks. At the end of the study, a significant decrease in FBS and HbA1c and an increase in INS sensitivity were observed [22]. In 2016, Mahdavi et al. prescribed 3 g/d of black seed oil to obese women for 8 weeks in a clinical trial. The results showed significant changes in levels in patients [14]. Also, Ansari et al. reported significant changes in FBS after 12 weeks by administering 2.5 cc/d of NS oil to diabetic patients with nephropathy [23]. According to the findings of their study, serum INS levels increased intergroup, although there was no significant difference compared to the placebo group. Because the primary route of INS excretion is through the urine, a decrease in urine volume in HD patients results in decreased INS excretion and thus increased blood INS levels [43]. However, previous research studies have indicated that NS enhances INS production. Due to the increasing influence of NS on urine volume in renal patients, more INS is probably eliminated in the urine. Hence, the intergroup differences in INS were not significant.

The NS can modulate blood sugar by various mechanisms. NS reduces carbohydrate digestion by inhibiting the intestinal alpha-glucosidase enzyme and reduces glucose absorption (like the mechanism of acarbose) [44,45]. Probably the main mechanism of the antidiabetic effect of NS is associated with stimulating insulin secretion. Insulin secretion is affected by a variety of factors. NS can increase insulin secretion by increasing GLP1 secretion [46,47], stimulating beta-adrenergic receptors [48], proliferating and regenerating pancreatic beta cells [49,50], and decreasing serum glucagon [51,52]. In addition, black seed can increase the sensitivity of insulin receptors by increasing the production of p-IRS and p-AKT and decreasing leptin secretion [32, 53]. It can also increase GLUT-4 synthesis [32] and stimulate key enzymes of glucose metabolism (including glucose-6-phosphate dehydrogenase (G6PDH), glucose-6-phosphatase (G6Pase), and fructose-1, 6-bisphosphatase (FBPase)) [54], which leads to increased glucose entry into the cells and consequently lower blood glucose levels.

According to the meta-analysis study by Mahmoodi et al., the most effective method of prescribing NS to reduce blood sugar is 2 g/d of its powder for at least 12 weeks [55]. However, since the amount of phosphorus in powder form is relatively high, the balance of serum phosphorus in renal patients is impaired. Therefore, to ensure no side effects and phosphorus accumulation, supplementation in the form of oil was performed.

## 5. Limitations

This clinical trial has some limitations. First, although in this study we tried to consider the confounding effect of the most important drugs used by patients in the final analysis (including calcium carbonate, calcitriol, sevelamer, and cinacalcet), due to the variety of drugs used in hemodialysis, several drugs were not included in the evaluations of this study. Second, to better understand NS's exact antidiabetic and anti-inflammatory effects, it is recommended to determine the C-peptide, glycated albumin, TNF-*α*, and glutathione. In this study, due to funding constraints, we failed to measure these indicators. Third, due to old age and memory impairment in dialysis patients, their adherence to diet and medication is not the same, which can cause problems in the results. However, through weekly follow-up, we tried to resolve this problem.

## 6. Conclusions

In conclusion, this study showed that supplementation with NS oil could reduce OS, inflammation, and blood sugar in diabetic patients undergoing HD. Our results are following previous studies. Considering the safety, low price, and many beneficial effects of NS on health, it can be prescribed as an adjunct treatment in diabetic HD patients.

## Figures and Tables

**Table 1:** Baseline characteristics of participants.

Variables	Nigella sativa group (*N* = 20)	Placebo group (*N* = 21)	*P* ^a^
Age (years)	49.60 (8.75)	48.57 (10.47)	.735^a^
BMI (kg/m^2^)	27.51 (4.03)	26.79 (2.94)	.517^a^
Weight (kg)	79.20 (12.55)	78.38 (10.99)	.825^a^
Duration of dialysis (years)	3.00 (2.00)	2.00 (1.50)	.138^b^
Hypertension disease (yes)	5 (25.0)	6 (28.6)	.796^c^
Sex			
Male	12 (60.0)	11 (52.4)	.623^c^
Female	8 (40.0)	10 (47.6)	
Use of drugs			
Calcium carbonate (yes)	19 (95.0)	15 (71.4)	.093^d^
Calcitriol (yes)	14 (70.0)	18 (85.7)	.277^d^
Sevelamer (yes)	14 (70.0)	10 (47.6)	.208^d^
Cinacalcet (yes)	2 (10.0)	4 (19.0)	.663^d^

Mean (SD) and median (IQR) are presented for normally and not normally distributed measures, respectively. Frequency (percentage within a subgroup) has been used for qualitative variables. BMI = body mass index; SD = standard deviation; IQR = interquartile range. The *P*-value for variable comparing between the two groups is calculated by^a^ independent sample *t*-test and,^b^ Mann-Whitney *U* test,^c^ Pearson's chi-square, and^d^ Fisher's exact test.

**Table 2:** Serum concentrations of oxidative stress markers, hs-CRP, and glycemic indices of the patients.

Variables	Nigella sativa group (*N* = 20)	Placebo group (*N* = 21)	MD	*P*
SOD (U/mL)				
Baseline	185.60 (4.50)	192.69 (4.75)	−7.09	.287^b^
End	203.48 (3.24)	198.03 (4.48)	5.44	.064^c^, .040^d^
MD (95% CI)	−17.88 (−26.87, −8.89)	-5.34 (-13.03, 2.33)		
P^a^	.001	.162		
TAC (mmol/L)				
Baseline	1.34 (.01)	1.36 (.01)	−.01	.331^b^
End	1.40 (.01)	1.35 (.01)	.05	<.001^c^, <.001^d^
MD (95% CI)	−.06 (−.08, −.04)	.01 (−.01, .03)		
P^a^	<.001	.353		
MDA (*μ*mol/L)				
Baseline	2.19 (.07)	1.91 (.06)	.27	.007^b^
End	1.86 (.05)	1.89 (.05)	−.02	.022^c^, .025^d^
MD (95% CI)	.32 (.23, .42)	.01 (-.11, .15)		
P^a^	<.001	.782		
Hs-CRP (mg/L)				
Baseline	8.59 (.40)	9.01 (.59)	−42	.560^b^
End	7.55 (.38)	8.86 (.57)	−1.30	.022^c^, .017^d^
MD (95% CI)	1.04 (.63, 1.44)	.15 (-.62, .93)		
P^a^	<.001	.680		
FBS (mg/dL)				
Baseline	190.70 (6.08)	156.57 (3.43)	3.24	.510^b^
End	149.91 (2.68)	152.85 (3.10)	−2.93	.004^c^, .027^d^
MD (95% CI)	−16.38 (−25.04, −7.72)	−1.99 (−13.68, 9.70)		
P^a^	.001	.726		
INS (*μ*IU/mL)				
Baseline	15.91 (2.07)	19.39 (2.49)	-3.47	.294^b^
End	19.66 (1.98)	20.00 (2.28)	-.34	.459^c^, .558^d^
MD (95% CI)	3.74 (1.10, 6,38)	.60 (−4.13, 5.35)		
P^a^	.008	.791		
HbA1c (%)				
Baseline	8.26 (.33)	8.38 (.37)	−11	.815^b^
End	7.76 (.23)	8.32 (.31)	−55	.009^c^, .014^d^
MD (95% CI)	−.49(−90, −09)	−.06 (−.29, .17)		
P^a^	.019	.572		

Mean (SD) and mean differences (95% CI) are presented for normally distrusted data; median (IQB) and median differences are presented for data not normally distributed. SOD = superoxide dismutase; MDA = malondialdehyde; TAC = total antioxidant capacity; hs-CRP = high-sensitivity C-reactive protein; FBS = fasting blood sugar; INS = insulin; MD = mean difference; SD = standard deviation; IQR = interquartile range; ANCOVA = analysis of covariance. P^a^ = paired samples *t*-test, P^b^ = independent samples *t*-test, P^c^ = ANCOVA test, adjusted for baseline values (Model 1), and P^d^ = ANCOVA test, adjusted for baseline values, energy intake^1^, and BMI (Model 2).

## Data Availability

The datasets generated during this study will be available via the corresponding author on a reasonable request.
